# Batch Crystallization of Xylitol by Cooling, Evaporative, and Antisolvent Crystallization

**DOI:** 10.1021/acs.cgd.2c01323

**Published:** 2023-01-26

**Authors:** Anna Zaykovskaya, Marjatta Louhi-Kultanen

**Affiliations:** Department of Chemical and Metallurgical Engineering, School of Chemical Engineering, Aalto University, PO Box 16100, Aalto, FI-02150Espoo, Finland

## Abstract

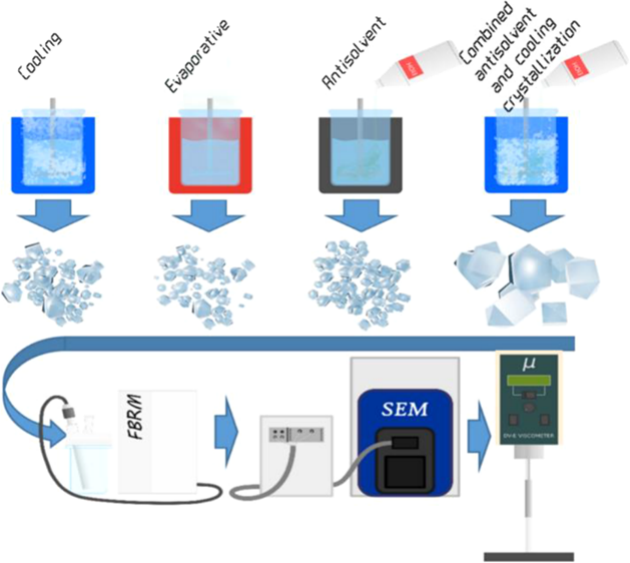

Four different techniques for xylitol crystallization, namely cooling, evaporative, antisolvent, and combined antisolvent and cooling crystallization, were investigated regarding their influence on the product crystal properties. Various batch times and mixing intensities were studied, and the antisolvent used was ethanol. Real-time monitoring of the count rates of various chord length fractions and distributions using focused beam reflectance measurement was conducted. Several solid characterization methods were used for studying the crystal size and shape, such as scanning electron microscopy and laser diffraction-based crystal size distribution analysis. Crystals ranging in size from 200 to 700 μm were obtained based on the analysis results by laser diffraction. The dynamic viscosity of saturated and undersaturated xylitol solution samples was measured; the density and refraction index were measured to determine the xylitol concentration in the mother liquor. Saturated xylitol solutions were found to have relatively high viscosities up to 129 mPa s in the studied temperature range. Viscosity can have a key role in crystallization kinetics, especially in cooling and evaporative crystallization. Mixing speed had a great influence, mainly on the secondary nucleation mechanism. The addition of ethanol decreased the viscosity, resulting in more uniform crystal shape and better filterability.

## Abstract 2

Batch cooling, evaporative, and antisolvent crystallization processes of xylitol were studied. The antisolvent studied was ethanol. High solution viscosity greatly reduced the crystallization rates. Higher mixing intensity decreased the crystal sizes in the case of thick suspensions.

## Introduction

Xylitol is a five-carbon sugar alcohol with applications in the food,^1^ pharmaceutical,^2^ ontological,^3^ and cosmetic industries.^4^ The increase in demand for xylitol in the food and pharmaceutical market has led to intensive research, with the aim of developing a cost-efficient xylitol production process. A low concentration of xylitol (<0.9%) naturally exists in fruits and vegetables, but its extraction from these sources is difficult and uneconomic.^5,6^ Xylitol can be obtained by a chemical process based on the reduction of xylose derived mainly from wood hydrolysates.^7^ Xylose reduction requires high temperature and pressure and an expensive catalyst.^8^ The biotechnological production of xylitol using yeast cells has been investigated as an alternative to the chemical process. Thus, different biomasses can be used as abundant and cheap feedstocks to produce xylitol from xylose by fermentation.^9−11^ Xylitol is conventionally recovered in solid form by crystallization from hydrolysis solutions. Crystallization is widely used for xylitol recovery because it allows pure polyol with solid consistency to be obtained from relatively impure solutions in a single step. Sugar alcohols are highly soluble in aqueous solutions, and their solubility increases at higher temperatures. As the xylitol solubility also varies at different temperatures, cooling crystallization can be considered as one of the potential crystallization methods. The crystallization methods investigated in the present work were batch cooling crystallization with or without the addition of an antisolvent, semi-batch antisolvent crystallization, combined semi-batch antisolvent crystallization and batch cooling crystallization, and semi-batch evaporative crystallization. Our objective was to compare the methodologies based on crystal yield and crystal properties by changing the cooling rate, residence time, solvent evaporation rate, antisolvent addition rate, and mixing intensity. Moreover, viscosity can have a key role in crystallization kinetics, especially in cooling and evaporative crystallization. In addition, mixing conditions usually affect crystallization, especially with high volume fractions of crystals and with long residence times. Changing the mixing conditions in a crystallizer can directly impact the kinetics of the crystallization process and the final crystal size.^12^

Xylitol production and crystallization have been the subjects of numerous studies^7,13−18^ which have focused on the biotechnological production of xylitol. Moreover, the influence of organic solvents and additives on the crystallization of sugars and sugar alcohols from aqueous solutions has been investigated in various papers.^16,17,19^ As a summary, the obtained results showed that alcohols, such as ethanol,^18,19^ methanol,^17^ and isopropanol,^11^ have the greatest influence on xylitol crystallization. However, the literature is devoid of data on the variations in crystallization methods and synthesis parameters: temperature, time, mixing conditions, and viscosity. Hence, an in-depth study of these crystallization parameters is necessary for crystallizing xylitol to deliver high crystallinity, suitable crystal morphology, and desired particle size distribution (PSD).

Thus, the present work focuses on investigating various crystallization methods for xylitol to achieve uniformity in terms of crystal size and shape. This is crucial, as the PSD and morphology significantly affect downstream processing, such as the filterability of the crystal mother liquor suspension, filter cake washing, and the drying properties of the crystal product.^20^

## Experimental Section

### Analysis of Mother Liquor

#### Viscosity Measurements of Initial Synthetic Xylitol Solutions

A Brookfield DV-E viscometer was used to measure the viscosity of xylitol solutions at the given shear rates and temperatures. The principle of operation of the DV-E viscometer is to rotate a spindle (immersed in the test fluid) through a calibrated spring. The viscous drag of the fluid against the spindle is measured by spring deflection. The spring deflection is measured with a rotary transducer which provides a torque signal. The measurement range of the DV-E instrument (in milliPascal seconds) is determined by the rotational speed of the spindle (10–100 rpm), the size and shape of the spindle, the container in which the spindle is rotating, and the full-scale torque of the calibrated spring.

#### Refractive Index Measurements of Aqueous and Ethanol-Containing Xylitol Solutions

Refractive index measurements were carried out with an Abbemat digital automatic refractometer. The measurement of the refractive index of the final mother liquor sample was used to determine the concentration of xylitol in the final solution. The samples obtained after crystallization were measured with a refractometer, and the exact concentration of xylitol in the mother liquor was determined based on the obtained calibration line. Eight solution samples were prepared by weighing xylitol and water to cover the required concentration range between 0 and 63 wt %. The refractive index values of these samples were measured at room temperature. The correlation coefficient based on the *R*-squared value of the obtained linear calibration line was 99.75%.

#### Liquid Density

The density of the saturated xylitol solutions was determined with an Anton Paar DMA 5000 M density meter. The measurements were performed to calculate the quantities of chemicals required for the set reactor volume.

### Crystallization of Xylitol

**Figure 1:**
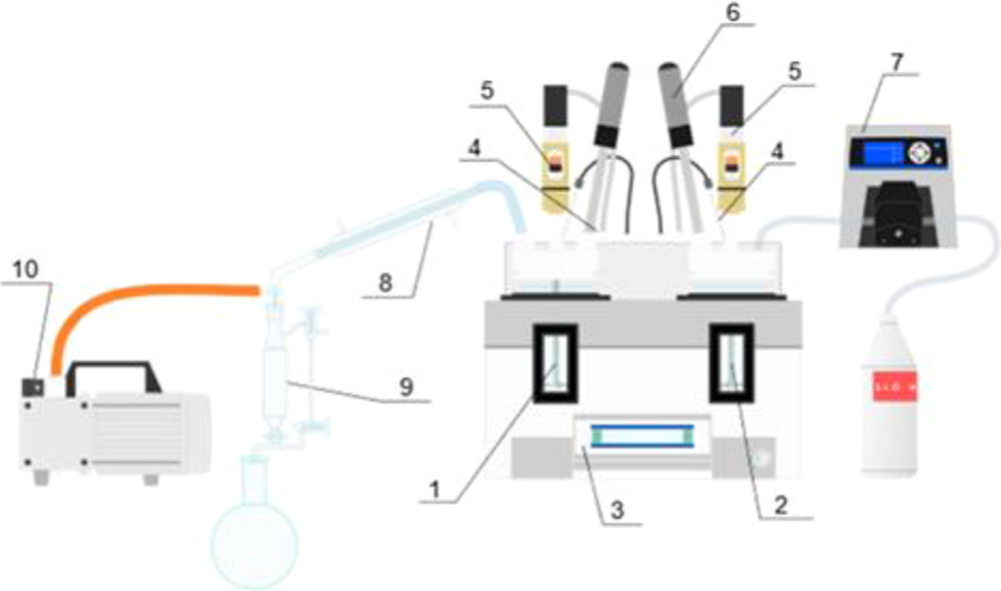
EasyMax 402 crystallization system. 1, 2–100 mL reactors; 3––control panel, 4––thermocouples, 5––stirrers, 6––FBRM, 7––peristaltic pump, 8––condenser, 9––liquid collector, 10––vacuum pump.

In the present work, four different techniques for xylitol crystallization and their operating parameters were evaluated regarding their influence on the product crystal properties: batch cooling, semi-batch evaporative, semi-batch antisolvent, and combined semi-batch antisolvent and batch cooling crystallization. A systematic study was carried out regarding the effect of crystallization parameters such as residence time, cooling rate, temperature, antisolvent content, and evaporation rate in an EasyMax 402 stirred reactor workstation by Mettler Toledo (Figure 1). An up-pumping pitched-blade four-blade stirrer with a diameter of 38 mm was used with the baffled 100 mL reactor. The mixing conditions were varied by changing the rotation speed to alter the dispersion degree of crystals in the reactor and to change the mass and heat transfer conditions. The mixing speed was 450 rpm or 800 rpm (tip speed of 0.90 and 1.59 m/s, respectively). In addition, a peristaltic pump connected to the crystallization system allowed a continuous supply of the antisolvent.

Due to the high solubility of xylitol, a certain amount of mother liquor that remained on the surface of the crystals after drying significantly increased the mass of the final crystals, leading to an overestimated quantification of the crystalline product. Therefore, the crystal mass was determined from the concentration difference between the initial and final solutions by refractive index measurements to avoid errors in determining the crystal quantity. Accurate concentration analysis is of importance for obtaining more precise crystal yield and *m*_obt_/*m*_th_ (obtained crystal mass/solubility-based theoretical crystal mass ×100%) calculations.

#### Cooling Crystallization

Aqueous saturated solutions were prepared for the studies on the batch cooling crystallization of xylitol (Sigma-Aldrich, ≥97.5%), based on the solubility data published by Wang et al.^21^ The temperature range between 10 and 50 °C and temperature differences of 10–15 °C were used. For instance, when a solution saturated at 40 °C was used, the temperature of the initial crystal-free solution was decreased from 40 to 25 °C at a constant cooling rate (0.5 K/min–0.5 K/h). Once the temperature reached 38.5 °C and the liquor became supersaturated, dry seed crystals were added through the reactor lid. The mass of added seeds was 1%^22^ of the theoretical crystal mass (*m*_th_), which was calculated from the theoretical solubility difference between 40 and 25 °C. A similar seeding procedure^22^ was used in the majority of xylitol crystallization studies. The crystal size distribution of the seed crystals was measured with a Malvern Mastersizer 2000, and the average size was 50 μm. Seed crystals were obtained by rapid cooling of the xylitol solution. During this series of experiments of cooling crystallization, various batch times were applied––from 0.5 to 48 h.

#### Semi-Batch Evaporative Crystallization

The second method investigated was semi-batch evaporative crystallization. It was performed in a vacuum, in 35 and 40 millibars and 30 and 40 °C, respectively, or a constant temperature of 50 °C and atmospheric pressure at 1 and 5 h. The evaporation rates were varied to change the supersaturation degree. Seed crystals were added in the beginning of the experiment, as shown in Figure 10.

#### Semi-Batch Antisolvent Crystallization

In the third method studied, based on semi-batch antisolvent crystallization utilizing ethanol (Altia Oyj, ≥99.5%), a certain amount of ethanol (15–40 g) was continuously pumped into an aqueous xylitol solution at a constant temperature of either 20 °C or 40 °C. The antisolvent was added to the solution surface where the supersaturation level can be relatively high due to the lower intensity of microscale mixing. The addition time of ethanol was altered to change the driving force, that is, the supersaturation degree. Saturated xylitol solutions were prepared based on the solubility data published by Wang et al.^21^ Seeding was performed 10 min after the beginning of the experiment to ensure that the solution was supersaturated.

#### Combined Semi-Batch Antisolvent Crystallization and Batch Cooling Crystallization

**Figure 2:**
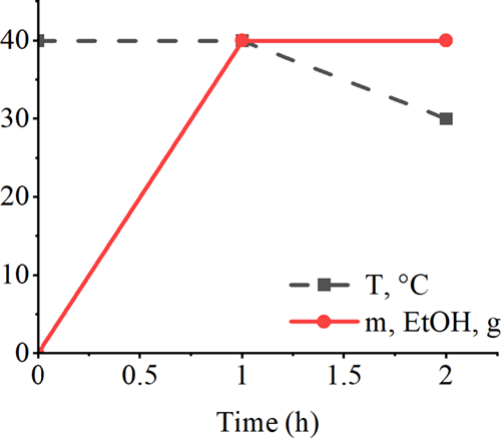
Periods of 2 h semi-batch antisolvent crystallization and batch cooling crystallization.

The fourth investigated technique was a combination of semi-batch antisolvent crystallization and batch cooling crystallization. The required amount of antisolvent (7–40 g) was continuously added to the reactor for 0.5–1 h, followed by cooling for 0.5–1 h. Seeding was made 10 min after the beginning of the experiment. Initial temperatures of 20 and 40 °C were used. The conducted experiment of combined antisolvent crystallization with ethanol addition and cooling crystallization is illustrated in Figure 2.

After the crystallization was complete, the crystallized material was washed first with an ethanol–water solution (85:15, wt %), then with pure ethanol, and dried in an oven at 55 °C.

### Characterization of Xylitol Crystals

#### Focused Beam Reflectance Measurement

During the crystallization experiments, an inline probe (Particle Track G400, Mettler Toledo) was immersed in the reactor and used to measure the count rates of various chord length size fractions to obtain kinetic data corresponding to nucleation and the crystal growth rate.

The Mettler-Toledo Particle Track G400 system is based on focused beam reflectance measurement (FBRM). A laser beam passes through a set of optics and focuses on a tight beam spot in the sapphire window. The optics rotates at a fixed speed of 2 m/s to scan the flow of particles through the window. The FBRM provides precise and highly sensitive chord length data collection to capture real-time changes. Before the experiments, the probe was cleaned and stabilized in advance with distilled water for zero particle counts. All the FBRM measurements were performed for chord length size fractions between 1 and 1000 μm with a time interval of 10 s.

#### Scanning Electron Microscopy

SEM was used to analyze the morphology, uniformity, and size of crystals. The main advantage of SEM is the possibility to observe solid-state topography with sufficient resolution. Measurements were carried out by using a table-top SEM TM4000 series from Hitachi High Technologies.

#### Particle Size Distribution Measurements

A Malvern Mastersizer 2000 system was used to analyze the PSD of the xylitol materials. The device can be applied for particles in the size range of 0.5–2000 μm.

## Determination of Mass Transfer Coefficient

The crystal growth rate depends usually on mass transfer and surface kinetics.^23^ In the case of high-viscous solutions, mass transfer can mainly dominate the crystal growth rate. Crystal growth rate, expressed by the mass of crystals forming over time, is directly dependent on the mass transfer coefficient, . Therefore, the mass transfer coefficient values were calculated to compare the studied saturated xylitol solutions. In the present work, mass transfer coefficient was determined using the Levins and Glastonbury equation:^24^
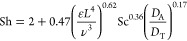
1where Sh = Sherwood number (*k*_L_*d/D*), ε = power input per unit mass of fluid (m^2^/s^3^), *L* = particle size (m), ν = kinematic viscosity (m^2^/s), Sc = Schmidt number (ν*/D*), *D*_A_ = diameter of the impeller (m), *D*_T_ = diameter of the tank (m), *k*_L_ = mass transfer coefficient (m/s), and *D* = diffusion coefficient (m^2^/s).

ε was calculated with the aid of VisiMix Laminar SV software for the performance of the crystallizer described in section ‘Crystallization of Xylitol’. Table A.5 in Supporting Information contains the data used in *k*_L_ calculations.

## Results and Discussion

### Viscosity Measurements of Pure and Ethanol-Containing Aqueous Xylitol Solutions

**Figure 3:**
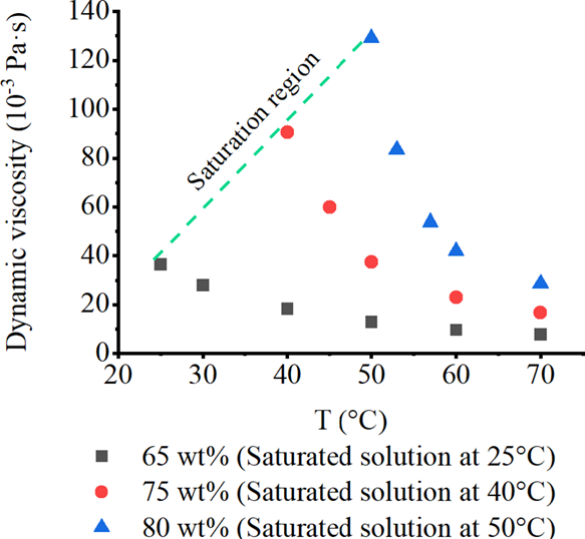
Dynamic viscosity of aqueous xylitol solutions.

Xylitol is highly soluble in water, and saturated xylitol solutions have relatively high viscosities, as shown in Figure 3. Viscosity measurements showed that the xylitol solutions were nearly Newtonian fluids as the viscosities did not vary significantly with the various studied shear rates (from 2.12 to 21.2 s^–1^) used in the viscosity measurements.

**Figure 4:**
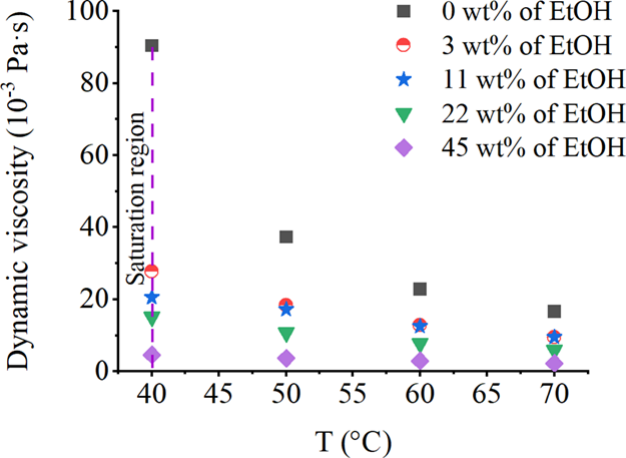
Dynamic viscosity of aqueous xylitol solution and ternary aqueous xylitol–ethanol solutions saturated at 40 °C.

In a higher temperature range, the overall crystallization rate is usually higher. Regarding the possible influence of viscosity on crystallization kinetics, it can be expected that when the system temperature decreases, the solution becomes more viscous and the solute less mobile in the solution. However, as shown in Figure 3, the dynamic viscosity of more concentrated xylitol solution saturated at a higher temperature is higher than the viscosity of solution saturated at a lower temperature. To select the appropriate temperature range for crystallization, Figure 3 type of viscosity data are useful. In antisolvent crystallization, the saturated solutions usually have lower viscosity (Figure 4) compared with the cooling and evaporative crystallization due to lower solubility levels and the presence of antisolvent.

### Crystallization Results

Xylitol crystals were produced by a systematic variation of operation parameters, such as residence time, temperature, cooling rate, mixing speed, antisolvent amount, and evaporation rate, resulting in crystalline products with specific crystal properties. The residence time was defined as the duration of the batch or semi-batch process. The total number of successful experiments was 35. The most promising crystallization conditions were chosen for further study and are summarized in Table A.1. The main criterion was to obtain the theoretical crystal yield. To initiate all the batch and semi-batch crystallization experiments in a controlled manner, seeding policy was followed based on the addition of 1 wt % seed crystals, with an average size of 50 μm, as discussed above.

#### Cooling Crystallization

**Figure 5:**
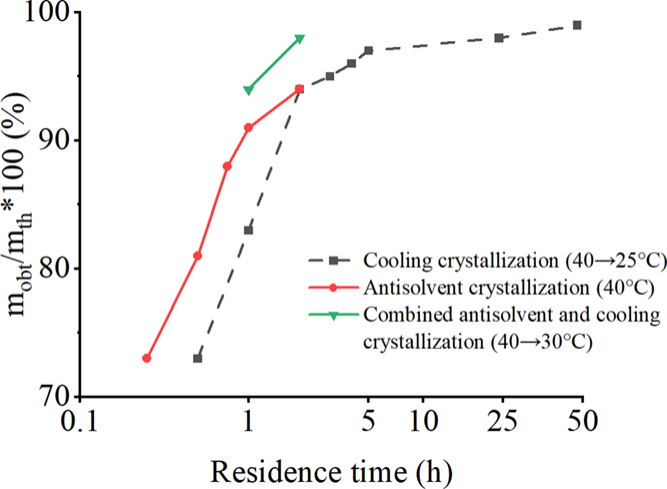
Effect of residence time on the final mass of xylitol crystals (450 rpm).

In the case of cooling crystallization, batch time, temperature, and cooling rate were varied. The cooling rates from 0.5 K/min to 0.5 K/h were applied. Based on the obtained data of *m*_obt_/*m*_th_, more than 90% was achieved with the batch time longer than 2 h. A cooling rate of 0.5 K/min (crystallization time of 30 min) reduced the *m*_obt_/*m*_th_ value to 73% (Figure 5). As can be seen from Figure 5, an increase in the batch time increased the final mass of the crystals. Increasing the batch time to 24 h or 48 h, with the cooling rate lower than 0.05 K/min, increased *m*_obt_/*m*_th_ only slightly. Therefore, the batch time of 2 h was concluded to be sufficient for batch cooling crystallization in the temperature range from 40 to 25 °C.

Additionally, in accordance with the obtained data, lower temperatures are preferable to get higher *m*_obt_/*m*_th_. This seems to be mainly due to the effect of viscosity on crystallization. The solubility of xylitol greatly increases with increasing temperature, and therefore solutions saturated at low temperatures are less viscous. For example, a solution saturated at 25 °C has a viscosity of 36.5 mPa s, compared to 129.1 mPa s at 50 °C (Figures 3 and 4).

#### Semi-Batch Evaporative Crystallization

In the case of evaporative crystallization, theoretical crystal mass, *m*_th_, was approached due to higher crystallization kinetics at lower temperatures. In addition, although evaporative crystallization in 5 h at atmospheric pressure at 50 °C led to the formation of crystals, this experiment required a lot of energy and time compared to other methods used, and therefore no further studies of evaporative crystallization at atmospheric pressure were carried out. Furthermore, reducing the crystallization time to 1 h led to the formation of a gel from which crystals could not be extracted. Moreover, higher dynamic viscosity also affected filterability by extending the filtration time significantly when crystals obtained by evaporative crystallization at 50 °C were filtered from the high-viscous mother liquor.

#### Semi-Batch Antisolvent Crystallization

In the case of antisolvent crystallization, the temperature change did not significantly affect the crystal yield, while the reaction time showed a similar trend as in the case of cooling and evaporative crystallization––with the increasing crystallization time, the theoretical yields were achieved more efficiently (Figure 5).

#### Combined Semi-Batch Antisolvent Crystallization and Batch Cooling Crystallization

In the case of combined semi-batch antisolvent crystallization and batch cooling crystallization, ethanol was added to the xylitol solution to reduce the viscosity of the liquid phase. In this type of crystallization, the same trend was observed: the longer the batch time, the more the crystals were obtained. For example, increasing the batch time from 1 to 2 h increased *m*_obt_/*m*_th_ from 94 to 98% (Figure 5). This method yielded the highest crystal yield of 72%, expressed by the ratio between the crystal mass and initial xylitol mass, and the highest *m*_obt_/*m*_th_ ratio of 98%. The addition of ethanol probably had a beneficial effect on cooling crystallization by reducing the viscosity and enhancing the heat and mass transfer. For instance, in experiment 7.C.4030.2h, the solution was 9 times more viscous than that in the experiment with ethanol addition, 14.AC.4030.2h (90 × 10^–3^ Pa s and 10 × 10^–3^ Pa s, respectively). In addition, using ethanol, it was possible to increase *m*_obt_/*m*_th_ from 93 to 98%. At the same time, it should be noted that for this type of combined antisolvent and cooling crystallization method, as well as for antisolvent crystallization, relatively high concentrations of ethanol are required (1 kg EtOH/1 kg xylitol crystals with an initial xylitol mass of 1.3 kg), which can increase greatly the solvent regeneration expenses and further the product price.

### Calculation Results of Mass Transfer Coefficient

**Figure 6:**
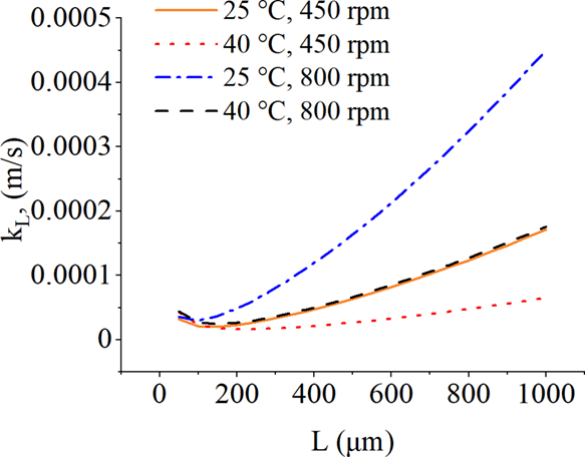
Dependence of the mass transfer coefficient on crystal size at 25 and 40 °C. The measured solution density and dynamic viscosity data of saturated solutions were used in the calculations. The diffusion coefficient data on xylitol in water reported by Winkelmann^25^ and the Sherwood number expression (eq 1) published by Levins and Glastonbury^24^ were used.

Figure 6 shows the obtained mass transfer coefficient values for the studied saturated solutions. High viscosity can reduce the mass transfer efficiency and further crystal growth kinetics, as shown in Table A.5 and Figure 6. Both viscosity and rotation speed have significant effects on *k*_L_ values. Furthermore, it can be concluded that the crystal growth rates of particles larger than 100–200 μm increase as a function of crystal size, whereas below this particle size range, declining trends of *k*_L_ values can be seen.

### Results of Crystal Characterization

Various physicochemical characterization methods were used, such as FBRM, SEM, and Malvern.

#### Focused Beam Reflectance Measurement

**Figure 7:**
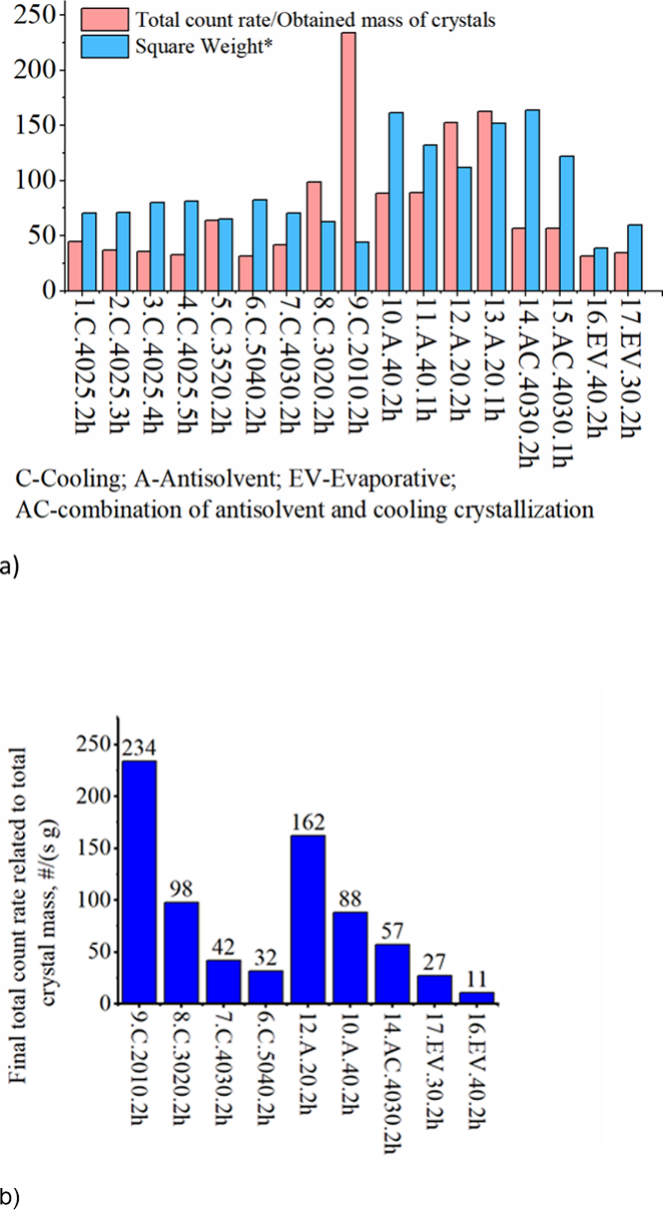
(a) Final total count rate related to total crystal mass (unit [#/(s g)]) and average chord length (unit [μm]) with 800 rpm. Square weight* refers to the length square weighted mean chord (square weight): the sum of the length square weighted counts per channel multiplied by the midpoint of that channel, divided by the sum of the length square weighted counts. (b) Total count rate per obtained crystal mass with the residence time of 2 h.

FBRM was used to monitor the real-time changes in the count rates of various chord length fractions and to collect the kinetic trends corresponding to nucleation and crystal growth rate. For the comparison of FBRM results obtained using different crystallization systems, the variation in crystal masses was taken into account by dividing the final total count rate by the crystal product mass. It was assumed that a higher total count rate per crystal mass indicates a higher nucleation rate. The results of FBRM at the end of each experiment are shown in Table A.2 and Figure 7a, and the total count rate per obtained crystal mass with the residence time of 2 h is shown in Figure 7b. It is likely that FBRM did not detect all the larger crystals properly as they tended to remain in the bottom part of the reactor. However, the FBRM and laser diffraction results of the end products are relatively consistent. In Figure 7 and below, coded references for experiments were used where the first character is the number of the experiment, the second character means the crystallization method (C––batch cooling crystallization, A––semi-batch antisolvent crystallization, AC––combined semi-batch antisolvent crystallization and batch cooling crystallization, and EV––semi-batch evaporative crystallization), the third is the temperature regime, and the last is the residence time of the experiment in hours.

**Figure 8:**
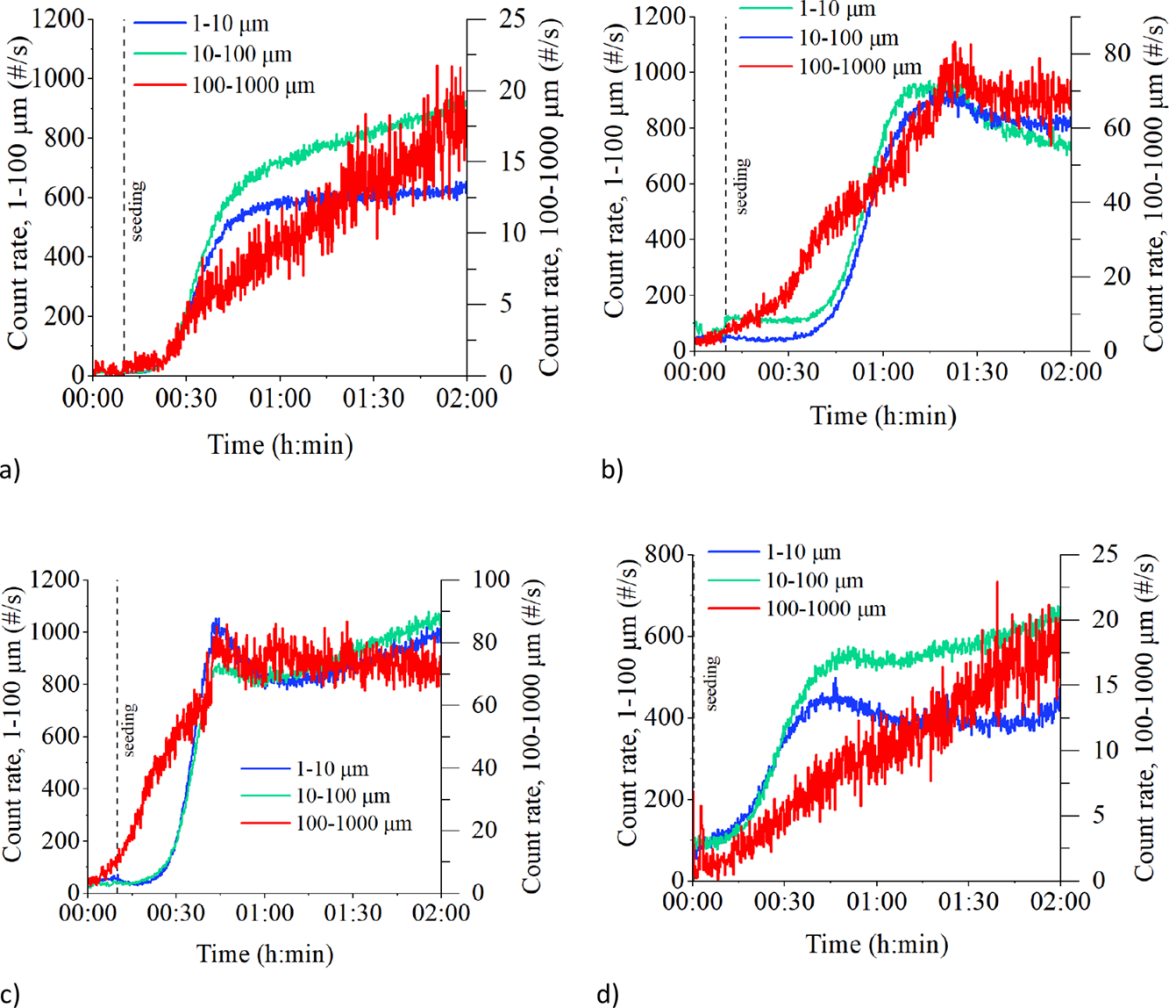
FBRM graphical data of (a) 2 h batch cooling crystallization between 40 and 25 °C in experiment 1.C.4025.2h with 450 rpm; (b) 2 h semi-batch antisolvent crystallization in experiment 10.A.40.2h with 450 rpm; (c) combined 1 h semi-batch antisolvent crystallization and 1 h batch cooling crystallization obtained in experiment 14.AC.4030.2h with 450 rpm; and (d) 2 h evaporative crystallization at 40 °C in experiment 16.EV.40.2h with 450 rpm.

As can be seen from Figure 7a and Table A.2, the chord lengths of xylitol particles obtained by cooling crystallization increased with an increase in crystallization time and higher temperature range. The obtained FBRM results in Figure 7b show that 9.C.2010.2h and 12.A.20.2h have the highest total count rate per crystal mass with the residence time of 2 h. In cooling crystallization, the total count rate per crystal mass decreased as a function of temperature, which indicates that nucleation rates are higher at lower temperatures. The lowest values of the total count rate per crystal mass were obtained by evaporative crystallization, whereas the presence of ethanol enhanced nucleation. As shown in Figure 7a, the nucleation rate is the highest in experiment 9.C.2010.2h, which is consistent with the smallest square weighted value corresponding to the average chord length. Moreover, as illustrated in Table A.1, experiment 9.C.2010.2h gave the smallest *m*_obt_/*m*_th_ with the residence time of 2 h. Thus, based on the obtained crystal quantity, it can be concluded that, during cooling crystallization, temperatures below 20 °C decreased crystallization and crystal growth rates, whereas the nucleation rate was relatively high based on the FBRM results. Furthermore, the addition of ethanol influenced positively the chord length, increasing it by 2 to 4 times. In the case of evaporative crystallization, the chord lengths were similar to those obtained by antisolvent crystallization. Figure 8 shows the obtained count rate tendencies of various chord length fractions for the studied crystallization methods. In addition, suspension densities varied from 120 to 410 g crystals/L in the mother liquor, depending on the applied crystallization method. The suspension density was the lowest in the case of the combination of semi-batch antisolvent and batch cooling crystallization. Secondary nucleation rates were the most significant with thicker suspensions, higher mixing intensities, and larger crystal sizes. In addition, Figure 7b indicates that the total count rates per crystal mass are higher at lower temperatures.

Figure 8 shows similarities in the increasing trends of the 100–1000 μm fraction in cooling and evaporative crystallization, whereas antisolvent crystallization with ethanol leads to the maximum values of count rates of the largest chord length fraction at 80 min in (b) and at 45 min during ethanol feeding in (c). Moreover, the count rates of 1–10 μm and 10–100 μm fractions are approximately in the same level in the presence of ethanol, while in cooling and evaporative crystallization, the count rates of the 10–100 μm fraction are higher than that of the 1–10 μm fraction.

#### SEM Analysis

**Figure 9:**
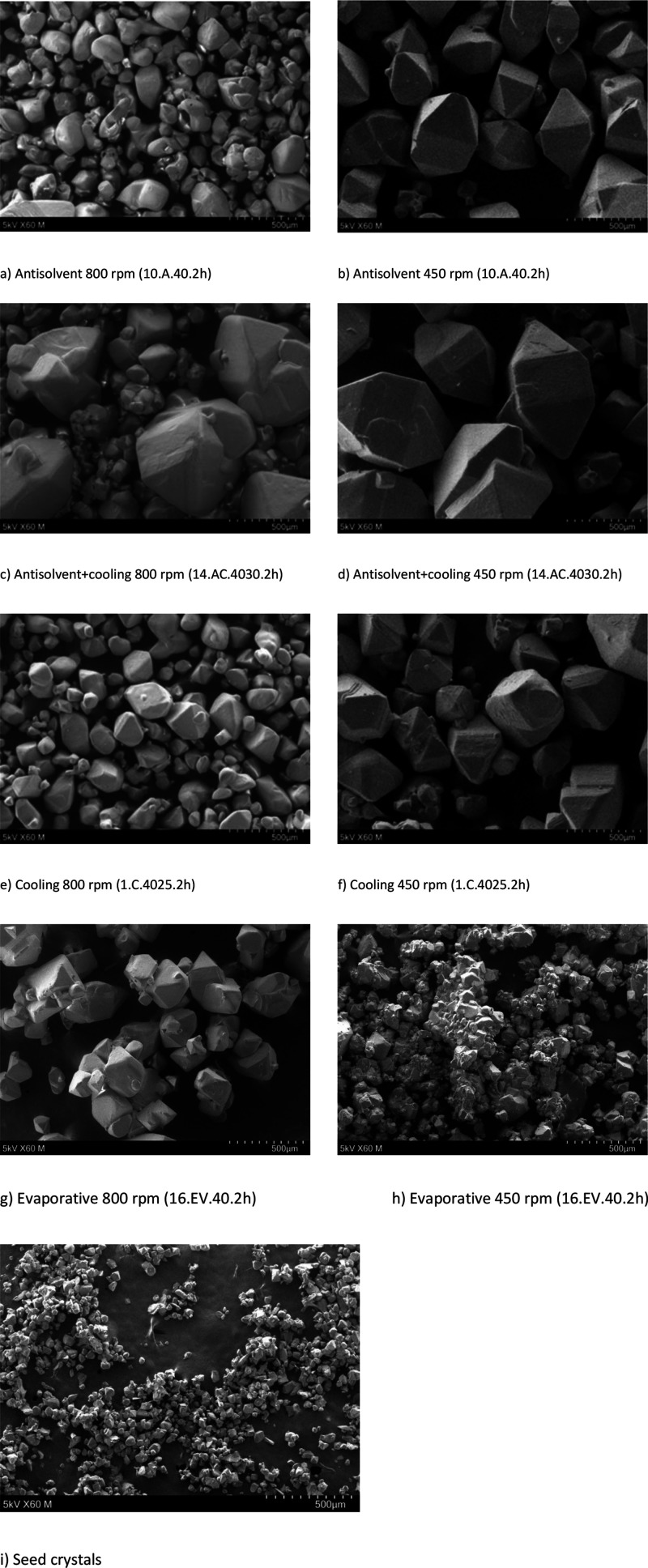
Typical SEM micrographs for the different crystallization methods and seed crystals.

SEM was used to analyze the morphology, uniformity, and size of crystals. The main advantage of SEM is the possibility to observe solid-state topography with a high resolution. Some of the micrographs obtained are shown in Figure 9. A magnification of 60× was used in all cases.

The SEM micrographs show that, when the antisolvent is added, xylitol crystals have a clearer elongated octahedral crystal shape and a larger crystal size. In addition, a lower mixing speed of 450 rpm gave regularly shaped, well-formed, homogeneous crystals in all cases, except evaporative crystallization. In the case of evaporative crystallization, the crystals varied in size and shape, which may be the result of the high viscosity of the mother liquor. Thus, with a higher mixing speed, the mass transfer has been enhanced, resulting in larger and more uniform crystals.

In the case of combined ethanol addition and cooling crystallization, a greater mixing intensity of 800 rpm resulted in the formation of agglomerates, which can adversely affect the further processing of crystals.

#### PSD Measurement Results

The PSD of the crystallized xylitol was analyzed by the laser diffraction analyzer, and the results are shown in Table A.3 and Figures A.2 and A.3.

Based on the obtained data, PSD varies significantly depending on the crystallization method applied and the specific parameters used during the crystallization process. Increasing the crystallization time and initial temperature led to an increase in the xylitol crystal size. Moreover, the addition of ethanol increased the PSD significantly. The same trends were observed in the chord length measurements by FBRM. To show the uniformity of crystals, a cumulative curve was plotted. The dependence of the crystallization method used on the PSD of xylitol is shown in Figure 8.

**Figure 10:**
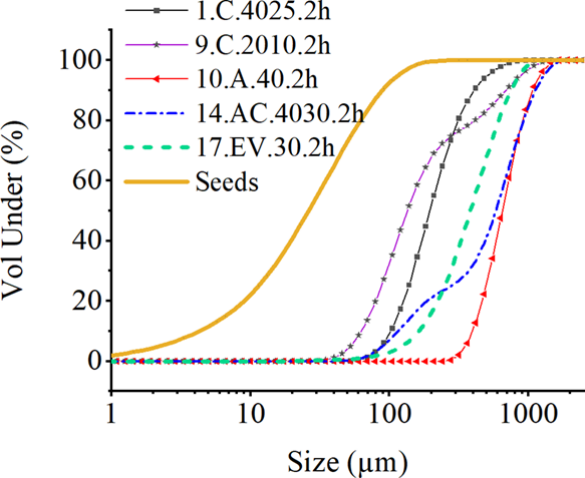
Cumulative particle size distribution curves obtained by laser diffraction analysis for xylitol samples generated by cooling, antisolvent, combined antisolvent and cooling, and evaporative crystallization with 800 rpm.

Cooling and antisolvent crystallization mostly resulted in uniform crystals. The only exception was experiment 9.C.2010.2h. As described above, during cooling crystallization, temperatures below 20 °C decreased the overall crystallization and crystal growth rates. Thus, agglomerates were also formed in the temperature range between 10 and 20 °C. Moreover, agglomeration occurred in experiment 14.AC.4030.2h. Thus, the combined antisolvent and cooling crystallization resulted in the best crystalline product with 450 rpm, whereas the mixing speed of 800 rpm was clearly too high.

Evaporative crystallization leads to the formation of agglomerates at lower mixing speeds, probably due to inefficient mass transfer and the high viscosity of the mother liquor at 30 and 40 °C.

**Figure 11:**
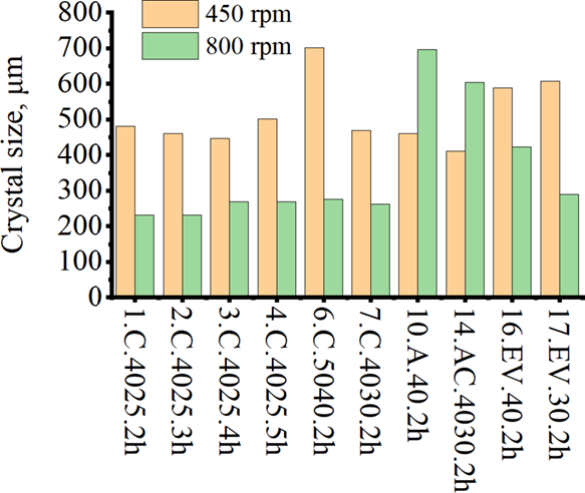
Dependence of the average crystal size (volume weighted mean L) obtained by laser diffraction analysis on the mixing speed.

Thus, the higher mixing speed decreased the size of the crystals in the case of cooling and increased it in the case of antisolvent addition and evaporative crystallization. Figure 11 demonstrates this dependence and illustrates the difference between the average crystal sizes obtained.

Figure 11 shows the dependence of the mixing speed on the final size of the xylitol crystals. The results are consistent with the SEM data. With an increase in the mixing speed from 450 to 800 rpm, the size of the crystals decreased significantly. This can be explained by the mechanical destruction of crystals, accompanied by the occurrence of secondary nucleation. At the same time, the PSD measurement results showed that increasing the stirring speed increased the crystal size of xylitol with the addition of ethanol. It is important to note that the laser diffraction analyzer measures the size of both single crystals and agglomerates. Therefore, in the case of experiments 14.AC.4030.2h, 16.EV.40.2h, and 17.EV.30.2h, the formation of agglomerates may have affected the final readings of the device. This can be proved by the SEM data as well as the plotted cumulative curve illustrated (Figure 11).

## Conclusions

The present work focused on the investigation of xylitol crystallization using various crystallization methods, that is, batch cooling, semi-batch evaporative, semi-batch antisolvent crystallization, and combined semi-batch antisolvent and batch cooling crystallization. One of the main aims was to investigate the effect of viscosity on crystallization, as xylitol is highly soluble in pure water. The dynamic viscosities of saturated pure aqueous xylitol solutions and ethanol-containing aqueous xylitol solutions were measured in the selected temperature range between 25 and 50 °C. The presence of ethanol enhanced the nucleation of xylitol in addition to its effect of reducing dynamic viscosity. According to the obtained FBRM results of cooling crystallization, the final total count rates per obtained crystal mass were higher at lower temperatures, which indicates that nucleation rates are higher at lower temperatures. In terms of the uniformity of the xylitol crystals produced, antisolvent crystallization with ethanol yielded the most favorable crystal properties with the moderate mixing speed. However, the relatively high ethanol requirement is an issue to be considered. A residence time of 2 h proved to be appropriate for three of the methods under the operational conditions used, with the exception of evaporative crystallization. In the case of large xylitol crystals and thick suspensions, higher mixing speeds increased the secondary nucleation rates greatly, which could be seen clearly from the results of count rates of various chord length fractions, crystal size distributions, and crystal micrographs. When comparing the temperature range between 10 and 50 °C in the cooling crystallization studies, batch cooling crystallization between 20 and 30 °C yielded the largest crystal sizes with the studied tip speed of 0.90 m/s.

## Supporting Information Available

Refractive index calibration line; PSD of the obtained xylitol crystals with the mixing speed of 450 rpm; PSD of the obtained xylitol crystals with the mixing speed of 800 rpm; dependence of the mass transfer coefficient on crystal length at 25 and 40 °C; crystallization conditions meeting the required crystal yields and properties; ; FBRM results with the mixing speed of 800 rpm; particle size distribution results obtained by the Malvern laser diffraction; supersaturation of the solutions at the seeding moment; and data used in the calculations of mass transfer coefficients; the particle size used in the calculations was 460 μm (PDF)

## Abbreviations

CR: cooling rate
FBRM: focused beam reflectance measurement
PSD: particle size distribution
SEM: scanning electron microscopy
