# Supplementary material for: Batch Crystallization of Xylitol by Cooling, Evaporative, and Antisolvent Crystallization
Source: Cryst Growth Des. 2023 Jan 26;23(3):1813–20. doi: 10.1021/acs.cgd.2c01323 (PMC9982812; doi:10.1021/acs.cgd.2c01323)
Supplement: Supplementary file 1 — cg2c01323_si_001.pdf [file cg2c01323_si_001.pdf]

Batch crystallization of xylitol by cooling, evaporative, and antisolvent crystallization

Anna Zaykovskaya[a,†], *Marjatta Louhi-Kultanen*[a,†*]

[a]Department of Chemical and Metallurgical Engineering, School of Chemical Engineering, Aalto University, PO Box 16100, FI-00076 AALTO (Espoo), Finland

[†]These authors contributed equally to this work

[*]Email: marjatta.louhi-kultanen@aalto.fi

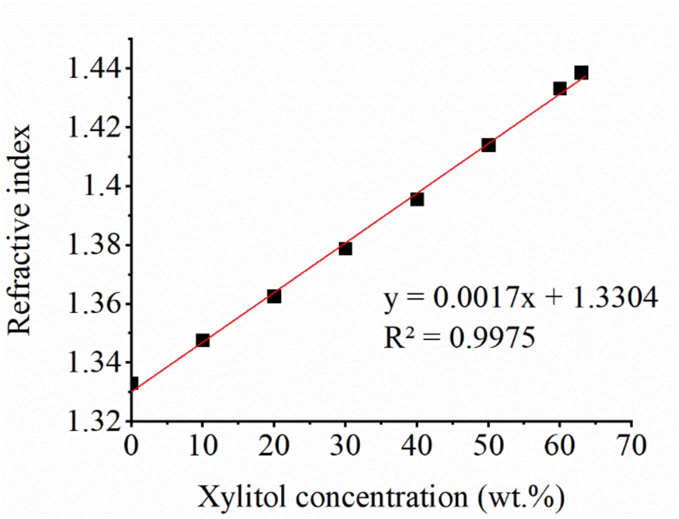

Figure A.1. Refractive index calibration line

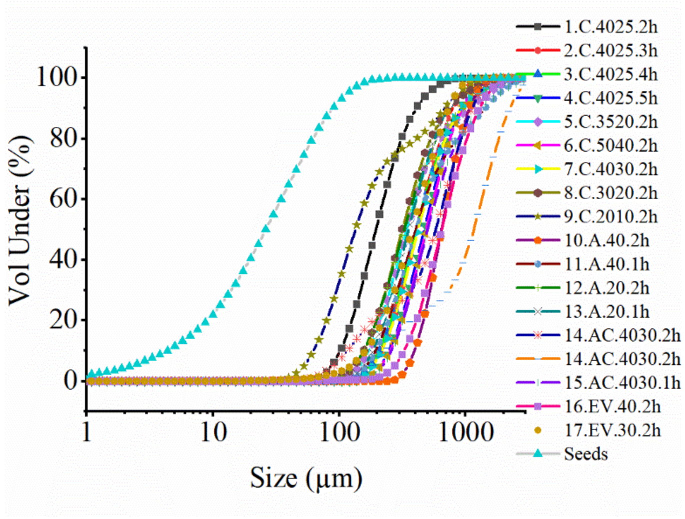

Figure A.2 PSD of seed crystals and product crystals obtained with the mixing speed of 800 rpm.

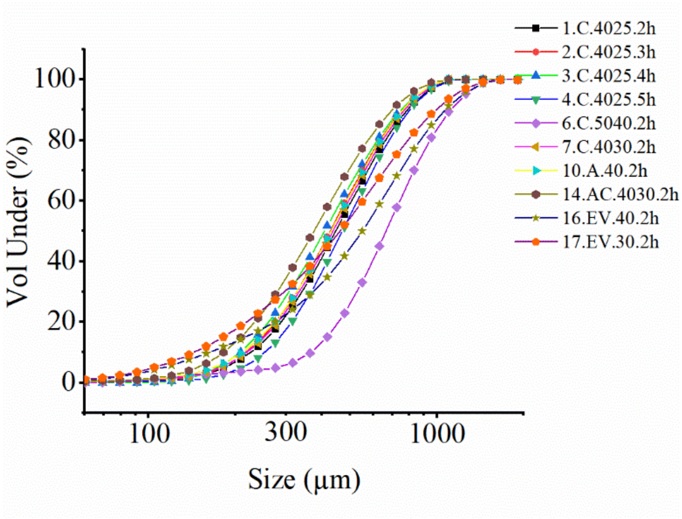

Figure A.3 PSD of xylitol crystals obtained with the mixing speed of 450 rpm.

Table A.1. Crystallization conditions meeting the required crystal yields and properties.

Ex#	Cr. Type	T, ˚C	m_x/ m_w (m_x/m_w/m_{et}), g/g or g/g/g (T*)	CR, K/min	m_{wr}, g	t, h	m_{th}, g	m_{obt}/m_{th}; m_{obt} wt%; g 800 rpm	m_{obt}/m_{th}; m_{obt} wt%; g 450 rpm
C.4025.0.5h				0.5		0.5		-	79
C.4025.1h				0.250		1		-	83
1.C.4025.2h		40→25	96.88/31.84 (40 °C)	0.125		2	37.46	94; 36	94; 36
2.C.4025.3h				0.083		3		95; 37	95; 37
3.C.4025.4h	Cool.			0.063		4		96; 37	95; 37
4.C.4025.5h				0.050		5		97; 38	96; 37
5.C.3520.2h		35→20	91.81/35.53 (35 °C)	0.125		2	33.60	96; 35	-
6.C.5040.2h		50→40	104.65/25.40 (50 °C)				27.36	91; 24	91; 24
7.C.4030.2h		40→30	96.88/31.84 (40 °C)	0.083		2	27.66	93; 27	92; 26
8.C.3020.2h		30→20	86.94/39.99 (30 °C)				21.41	94; 23	-
9.C.2010.2h		20→10	77.29/47.16 (20 °C)				18.46	89; 21	-
10.A.40.2h	Antis.	40	51.51/17.18/40.05 (40 °C)	-		2	20.38	93; 37	91
11.A.40.1h						1		91; 36	-

12.A.20.2h		20	41.68/25.44/39.13 (20 °C)	-		2	19.98	94; 45	-
13.A.20.1h				-		1		91; 44	-
14.AC.4030.2h	Antis+ Cool.	40→30	51.51/17.18/40.05 (40 °C)	0.167		2	38.09	98; 72	>99
15.AC.4030.1h				0.333		1		94; 70	-
16.EV.40.2h	Evap. Vac.	40	96.88/31.84 (40 °C)	-	15.40 (48%)	2	50.02	97; 52	-
17.EV.30.2h		30	86.94/39.99 (30 °C)	-	20.14 (50%)	2	43.15	97; 50	-

m_x – mass of xylitol, m_w – mass of water, m_{et} – mass of ethanol, CR – Cooling rate, T* - equilibrium temperature, m_{wr} mass of water removed by evaporation, m_{th} - theoretical crystal mass, m_{obt} – obtained crystal mass.

Table A.2. FBRM measurement results with the mixing speed of 800 rpm.

Ex#	Cr. Type	Count rate 1-10 µm, #/s	Count rate 10-100 µm, #/s	Count rate 100-1000 µm, #/s	m_{obt}/V g/L	Total count rate/m_{obt} #/(s g)	Square weight* µm
1.C.4025.2h	Cool.	634.07	928.65	19.85	352.12	44.94	70.30
2.C.4025.3h		518.63	786.36	19.13	355.87	37.21	71.30
3.C.4025.4h		464.29	793.67	22.14	359.62	35.97	80.30
4.C.4025.5h		406.35	761.34	28.56	363.36	32.92	81.23
5.C.3520.2h		944.14	1097.86	14.57	322.56	63.76	65.19
6.C.5040.2h		287.40	485.96	19.58	248.98	31.85	82.39
7.C.4030.2h		419.03	635.06	14.63	257.24	41.55	70.71
8.C.3020.2h		973.52	993.67	12.92	201.25	98.39	62.71
9.C.2010.2h		2218.91	1575.02	3.27	164.29	**233.75****	44.40
10.A.40.2h	Antis.	749.37	835.23	69.91	189.53	88.24	**161.41*****
11.A.40.1h		681.68	881.23	74.55	185.46	89.27	132.35
12.A.20.2h		1373.63	1396.11	62.12	187.81	**152.40****	112.03
13.A.20.1h		1469.72	1421.61	62.81	181.82	162.48	151.99
14.AC.4030.2h	Antis.+ Cool.	1007.33	1043.94	67.59	373.28	56.76	**163.90*****
15.AC.4030.1h		887.42	1054.04	82.70	358.05	56.53	122.19
16.EV.40.2h	Evap. Vac.	193.03	330.07	26.11	485.19	11.32	106.91
17.EV.30.2h		460.56	651.14	19.77	418.56	27.03	82.56

*Length Square Weighted Mean Chord (Square Weight) – The sum of the length square weighted counts per channel multiplied by the midpoint of that channel, divided by the sum of the length square weighted counts.

**Highest total count rates per crystal mass obtained with residence time of 2 h

***Largest Length Square Weighted Mean Chord

Table A.3. Particle size distribution results obtained by Malvern laser diffraction.

Ex#	Crystallization type	T, °C	Crystal size: average ⁄ d(0.5), μm, 800 rpm	Crystal size: average ⁄ d(0.5), μm, 450 rpm
C.4025.0.5h	Cooling	40→25	-	216/190
C.4025.1h			-	406⁄372
1.C.4025.2h			231/195	480/447
2.C.4025.3h			232/205	460/427
3.C.4025.4h			268/203	447/408
4.C.4025.5h			269/236	502/471
5.C.3520.2h		35→20	246/171	-
6.C.5040.2h		50→40	275/244	702/666
7.C.4030.2h		40→30	262/212	468/437
8.C.3020.2h		30→20	201/158	-
9.C.2010.2h		20→10	254/134	-
10.A.40.2h	Antisolvent	40	696/647	460/430
11.A.40.1h			306/199	-
12.A.20.2h		20	228/163	-
13.A.20.1h			221/178	-
14.AC.4030.2h	Combined Antisolvent and Cooling	40→30	603/582	-
15.AC.4030.1h			340/251	-
16.EV.40.2h	Evaporative under Vacuum	40	423/382	-
17.EV.30.2h		30	290/257	-

Table A.4. Supersaturation of the solutions at the seeding moment.

#	c (g/kg H_2O)	c*(g/kg H_2O)	Δc (c-c*, g/kg H_2O)	S (c/c*, -)	σ [(c-c*)/c*, -]
1.C.4025.2h	3000	2836	164	1.06	0.06
2.C.4025.3h	3000	2836	164	1.06	0.06
3.C.4025.4h	3000	2836	164	1.06	0.06
4.C.4025.5h	3000	2836	164	1.06	0.06
5.C.3520.2h	2558	2450	108	1.04	0.04
6.C.5040.2h	4070	3877	193	1.05	0.05
7.C.4030.2h	3000	2836	164	1.06	0.06
8.C.3020.2h	2222	2133	89	1.04	0.04
9.C.2010.2h	1711	1647	64	1.04	0.04
10.A.40.2h	3000	2267	733	1.32	0.32
11.A.40.1h	3000	1907	1093	1.57	0.57
12.A.20.2h	1703	1208	495	1.41	0.41
13.A.20.1h	1703	960	743	1.77	0.77
14.AC.4030.2h	3000	2103	897	1.43	0.43
15.AC.4030.1h	3000	2103	897	1.43	0.43
16.EV.40.2h	3000	3000	0	1	0
17.EV.30.2h	2221	2222	0	1	0

Table A.5. Data used in calculations of mass transfer coefficient. The particle size used in calculations was 460 µm.

T, K	ρ, kg/m³	µ, Pa s	ν, m²/s	Re_{mixer}	N_p	P, W	ε, W/kg	D, m²/s*	$D_{A,}$ m	$D_{T,}$ m	Sh	k_L , m/s
450 rpm												
298	1249	0.0365	2.92×10^{-5}	370.7	2.92	0.12	1.219	7.58×10^{-10}	3.8×10^{-2}	5.0×10^{-2}	34.64	5.7×10^{-5}
313	1287	0.0905	7.03×10^{-5}	154.1	3.40	0.15	1.463	1.06×10^{-9}	3.8×10^{-2}	5.0×10^{-2}	10.68	2.5×10^{-5}
800 rpm												
298	1249	0.0365	2.92×10^{-5}	659.0	2.5	0.58	5.841	7.58×10^{-10}	3.8×10^{-2}	5.0×10^{-2}	17.45	5.8×10^{-5}
313	1287	0.0905	7.03×10^{-5}	273.9	3.1	0.75	7.493	1.06×10^{-9}	3.8×10^{-2}	5.0×10^{-2}	6.28	2.9×10^{-5}

* Winkelmann[25]
